# 1233. Serious Toxicities During Antimicrobial Therapy for Disseminated Nocardia Infection in Solid Organ Transplant Recipients

**DOI:** 10.1093/ofid/ofab466.1425

**Published:** 2021-12-04

**Authors:** Christopher Saling, Sabirah N Kasule, Holenarasipur R Vikram

**Affiliations:** 1 Mayo Clinic in Arizona, PHOENIX, Arizona; 2 Mayo Clinic hospital, Phoenix, Arizona, Phoenix, AZ

## Abstract

**Background:**

Management of disseminated *Nocardia* (NC) infection in transplant recipients requires prolonged antimicrobial therapy. Treatment can be particularly challenging if NC is resistant to standard agents. Drug toxicities can further limit options. We present a series of transplant patients with multi-drug resistant, disseminated NC infection complicated by serious adverse reactions to sequential antimicrobials.

**Methods:**

This is a prospective review monitoring response to treatment of disseminated NC as well as adverse events to therapies.

**Results:**

The first case is a 66-year old heart transplant patient who presented with fever and cough. Investigations revealed *N. otitidiscaviarum* lung lesion and multiple brain abscesses. Trimethoprim-sulfamethoxazole (TMP-SMX) and linezolid were started empirically. NC was fully susceptible to linezolid only, and intermediate to quinolones and tobramycin. Linezolid was switched to ciprofloxacin due to ongoing cytopenia, and dose of TMP-SMX was reduced due to renal insufficiency. Repeat brain MRI showed enlarging abscesses; regimen was changed to linezolid and moxifloxacin. Severe peripheral neuropathy led to linezolid discontinuation and initiation of high-dose doxycycline plus moxifloxacin. One year into therapy, he presented with a large aortic dissection. His long-term quinolone therapy was felt to be contributory. He underwent aortic stent placement and remains on doxycycline monotherapy. The second case is a 74-year old female renal transplant patient who presented with fevers. A perinephric abscess was found which grew *N. farcinica* resistant to floroquinolones and clarithromycin, and intermediate to doxycycline. Further imaging also revealed pulmonary and brain involvement. TMP-SMX was started but soon switched to linezolid due to acute kidney injury. One month later she presented with severe thrombocytopenia and subdural hematoma thought to be secondary to linezolid. She died despite surgery.

**Conclusion:**

This series illustrates challenges encountered in the treatment of disseminated NC infection in transplant recipients. Multidrug resistant NC coupled with serious toxicities of therapies often severely limits treatment options. Counseling patients and closely monitoring for adverse events is essential.

**Disclosures:**

**All Authors**: No reported disclosures

